# Supplementary material for: Bayesian multistate models for measuring invasive carp movement and evaluating telemetry array performance
Source: PeerJ. 2024 Aug 6;12:e17834. doi: 10.7717/peerj.17834 (PMC11313411; doi:10.7717/peerj.17834)
Supplement: Supplemental Information 1 — Model included the effect of species, the river pool fish were tagged in, the interaction between pool and species, and included a random effect for tagging year to control for possible between-year differences. The model used a used a negative binomial distribution due to over-dispersion of count data. Pr(¿—z—) values ¡0.05 bolded. [file peerj-12-17834-s001.docx]

|  | | | | |
| --- | --- | --- | --- | --- |
| **Model: Number dates detected ~ River pool + Species + River pool * Species + (1 \| ReleaseYear)** | | | | |
|  | Estimate | Standard error | z value | Pr(>\|z\|) |
| Intercept | 3.419 | 0.383 | 8.925 | **< 2e-16** |
| Pool - La Grange | -0.626 | 0.452 | -1.386 | 0.165634 |
| Pool - Peoria | -2.419 | 0.656 | -3.686 | **0.000228** |
| Pool - Starved Rock | 0.601 | 0.378 | 1.592 | 0.111361 |
| Pool - Marseilles | 1.443 | 0.368 | 3.922 | **0.0000879** |
| Pool - Dresden Island | 1.866 | 0.354 | 5.265 | **0.00000014** |
| Species – silver carp | -0.659 | 0.551 | -1.195 | 0.232034 |
| La Grange * silver carp | 0.738 | 0.683 | 1.080 | 0.280173 |
| Peoria * silver carp | 2.861 | 0.806 | 3.549 | **0.000387** |
| Starved Rock * silver carp | 1.536 | 0.588 | 2.612 | **0.008997** |
| Marseilles * silver carp | 0.073 | 0.581 | 0.126 | 0.899462 |
| Dresden Island x silver carp | 0.522 | 0.586 | 0.890 | 0.37331 |
|  | Random effects (variance) | | | |
| Release Year | 0.2652 |  |  |  |
