# Supplementary material for: Bayesian multistate models for measuring invasive carp movement and evaluating telemetry array performance
Source: PeerJ. 2024 Aug 6;12:e17834. doi: 10.7717/peerj.17834 (PMC11313411; doi:10.7717/peerj.17834)
Supplement: Supplemental Information 2 — Model included the effect of species, the river pool fish were tagged in, the interaction between pool and species, and included a random effect for tagging year to control for possible between-year differences. The model used a used a negative binomial distribution due to over-dispersion of count data. Pr(¿—z—) values ¡0.05 bolded. [file peerj-12-17834-s002.docx]

|  | | | | |
| --- | --- | --- | --- | --- |
| **Model: Detection period in days ~ River pool + Species + River pool * Species + (1 \| ReleaseYear)** | | | | |
|  | Estimate | Standard error | z value | Pr(>\|z\|) |
| Intercept | 4.833 | 0.357 | 13.544 | **< 2e-16** |
| Pool - La Grange | 0.113 | 0.408 | 0.277 | 0.7818 |
| Pool - Peoria | -1.411 | 0.540 | -2.615 | **0.00891** |
| Pool - Starved Rock | 0.611 | 0.346 | 1.766 | 0.07733 |
| Pool - Marseilles | 1.323 | 0.330 | 4.005 | **0.0000619** |
| Pool - Dresden Island | 1.634 | 0.319 | 5.119 | **0.000000306** |
| Species – silver carp | -0.736 | 0.490 | -1.503 | 0.13289 |
| La Grange * silver carp | -0.190 | 0.615 | -0.309 | 0.7575 |
| Peoria * silver carp | 2.745 | 0.682 | 4.027 | **0.0000564** |
| Starved Rock * silver carp | 0.952 | 0.524 | 1.816 | 0.06938 |
| Marseilles * silver carp | 0.520 | 0.519 | 1.002 | 0.3164 |
| Dresden Island x silver carp | 0.492 | 0.523 | 0.941 | 0.34655 |
|  | Random effects (variance) | | | |
| Release Year | 0.2855 |  |  |  |
