# Supplementary figures and images for: Bayesian multistate models for measuring invasive carp movement and evaluating telemetry array performance
Source: PeerJ. 2024 Aug 6;12:e17834. doi: 10.7717/peerj.17834 (PMC11313411; doi:10.7717/peerj.17834)

SC-138

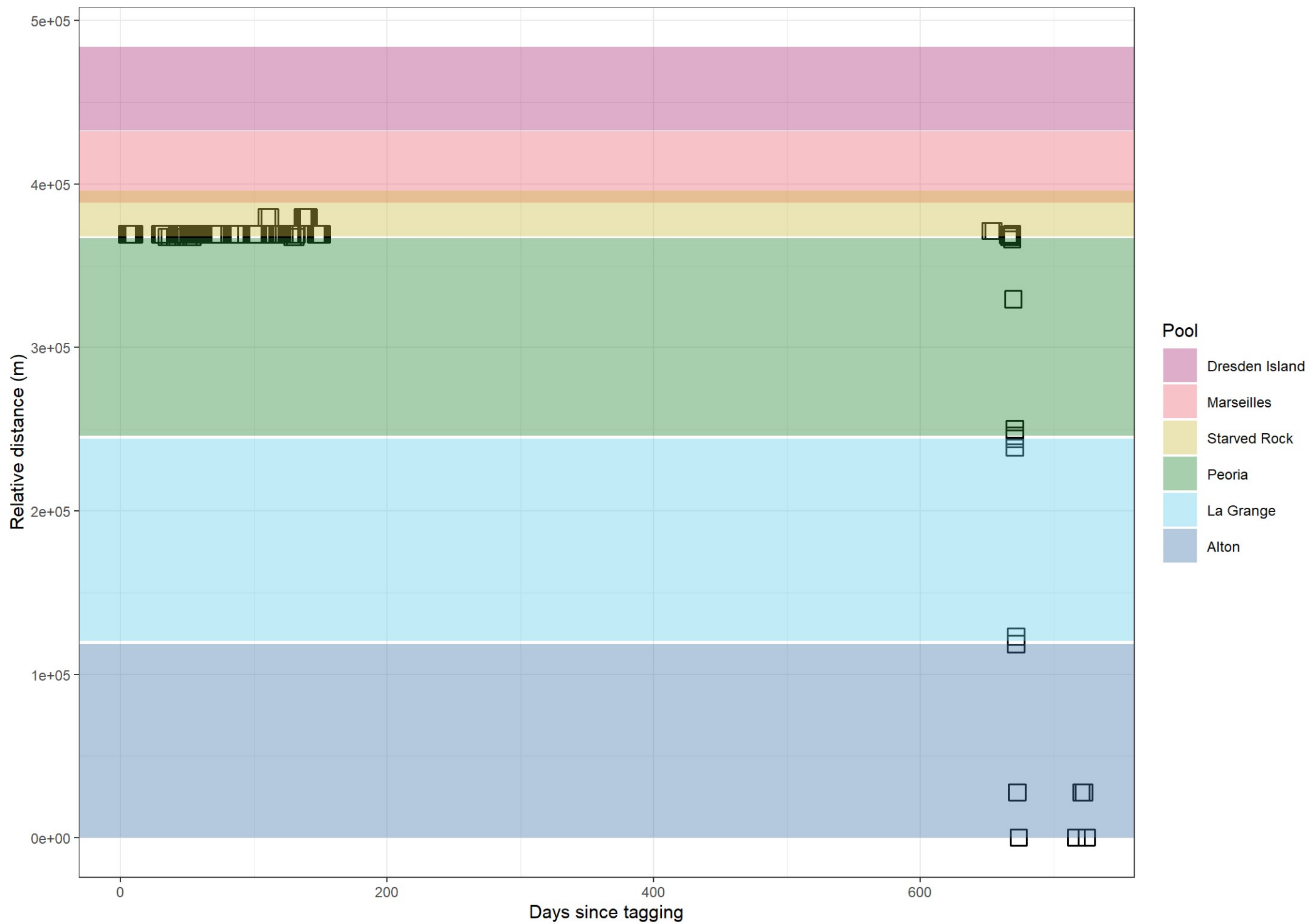

Supplement: Supplemental Information 3 — Each square signifies at least one detection per receiver per day since the day the fish was tagged. Receiver locations are shown on the vertical axis by the approximate distance in meters from the confluence of the Illinois River with the Mississippi River. The fish in this example demonstrates both up and downstream movements though Starved Rock, Peoria, La Grange, and Alton pools. However, after summarizing data to a monthly timestep, only a downstream movement from Starved Rock pool to Alton pool was used in the multistate model. [file peerj-12-17834-s003.pdf]
