# Supplementary material for: Bayesian multistate models for measuring invasive carp movement and evaluating telemetry array performance
Source: PeerJ. 2024 Aug 6;12:e17834. doi: 10.7717/peerj.17834 (PMC11313411; doi:10.7717/peerj.17834)
Supplement: Supplemental Information 4 — Total number of observations in each Illinois River pool (including release pool) for each species used in the Bayesian multistate model after summarizing to a monthly timestep. [file peerj-12-17834-s004.docx]

| Species | Alton | La Grange | Peoria | Starved Rock | Marseilles | Dresden Island |
| --- | --- | --- | --- | --- | --- | --- |
| Silver Carp | 19 | 64 | 287 | 1442 | 828 | 355 |
| Bighead Carp | 35 | 37 | 39 | 212 | 225 | 786 |
