# Supplementary material for: Bayesian multistate models for measuring invasive carp movement and evaluating telemetry array performance
Source: PeerJ. 2024 Aug 6;12:e17834. doi: 10.7717/peerj.17834 (PMC11313411; doi:10.7717/peerj.17834)
Supplement: Supplemental Information 5 — Parameter estimates are presented as median values (95% credible intervals) sampled from the posterior distributions. Illinois River pools included in the model were Alton (A), La Grange (L), Peoria (P), Starved Rock (S), Marseilles (M), and Dresden Island (D). Pool to pool movement was the product of the transition probability, survival probability, and battery life. Probability of detection was modeled as a logistic function dependent on the mean number of receivers deployed with a random effect for pool. [file peerj-12-17834-s005.docx]

| Model Parameter | From | To | Silver Carp | Bighead Carp |
| --- | --- | --- | --- | --- |
| transition probability | A | D | 0.0527 (0.0345-0.2110) | 0.1433 (0.0000–0.1858) |
|  | A | M | 0.1493 (0.0000‑0.1899) | 0.0001 (0.0000–0.1542) |
|  | A | S | 0.0000 (0.0000–0.0056) | 0.0000 (0.0000–0.0056) |
|  | A | P | 0.0000 (0.0000–0.0041) | 0.0000 (0.0000–0.0102) |
|  | A | L | 0.0029 (0.0000–0.0444) | 0.0151 (0.0034–0.0359) |
|  | A | A | 0.7954 (0.7410–0.8706) | 0.8342 (0.7895–0.8849) |
|  | L | D | 0.0000 (0.0000–0.0007) | 0.0000 (0.0000–0.0029) |
|  | L | M | 0.0000 (0.0000–0.1113) | 0.1266 (0.0000–0.1694) |
|  | L | S | 0.0393 (0.0000–0.0606) | 0.0000 (0.0000–0.0090) |
|  | L | P | 0.0564 (0.0212–0.0841) | 0.0050 (0.0000–0.0396) |
|  | L | L | 0.8819 (0.8440–0.9130) | 0.8295 (0.6773–0.8828) |
|  | L | A | 0.0071 (0.0026–0.0152) | 0.0272 (0.0111–0.3034) |
|  | P | D | 0.0000 (0.0000–0.0017) | 0.0001 (0.0000–0.1349) |
|  | P | M | 0.0000 (0.0000–0.0016) | 0.0000 (0.0000–0.0070) |
|  | P | S | 0.0277 (0.0080–0.0998) | 0.0880 (0.0160–0.1489) |
|  | P | P | 0.6368 (0.5215–0.8538) | 0.8579 (0.8071–0.9124) |
|  | P | L | 0.3404 (0.0727–0.4562) | 0.0186 (0.0024–0.0642) |
|  | P | A | 0.0000 (0.0000–0.0031) | 0.0000 (0.0000–0.0087) |
|  | S | D | 0.0000 (0.0000–0.0005) | 0.0000 (0.0000–0.0037) |
|  | S | M | 0.0000 (0.0000–0.0005) | 0.0207 (0.0063–0.0467) |
|  | S | S | 0.9156 (0.8945–0.9347) | 0.7404 (0.6475–0.8324) |
|  | S | P | 0.0310 (0.0186–0.0830) | 0.2186 (0.1355–0.3107) |
|  | S | L | 0.0541 (0.0019–0.0768) | 0.0120 (0.0004–0.0530) |
|  | S | A | 0.0000 (0.0000–0.0021) | 0.0000 (0.0000–0.0086) |
|  | M | D | 0.0009 (0.0000–0.0047) | 0.0163 (0.0078–0.0294) |
|  | M | M | 0.7680 (0.7229–0.8546) | 0.7696 (0.7294–0.8078) |
|  | M | S | 0.0104 (0.0044–0.0197) | 0.0209 (0.0107–0.0359) |
|  | M | P | 0.0012 (0.0001–0.0064) | 0.0000 (0.0000–0.0039) |
|  | M | L | 0.0077 (0.0003–0.2244) | 0.1786 (0.0000–0.2265) |
|  | M | A | 0.2095 (0.0000–0.2574) | 0.0001 (0.0000–0.2192) |
|  | D | D | 0.8473 (0.8047–0.8845) | 0.8089 (0.7791–0.8366) |
|  | D | M | 0.0110 (0.0033–0.0261) | 0.0182 (0.0102–0.0294) |
|  | D | S | 0.0000 (0.0000–0.0024) | 0.0023 (0.0003–0.0074) |
|  | D | P | 0.0000 (0.0000–0.0024) | 0.0081 (0.0016–0.1896) |
|  | D | L | 0.0000 (0.0000–0.0036) | 0.0000 (0.0000–0.0039) |
|  | D | A | 0.1399 (0.1041–0.1815) | 0.1534 (0.0000–0.1887) |
| survival probability |  |  | 0.9634 (0.9556–0.9708) | 0.9690 (0.9611–0.9762) |
| battery life |  |  | 0.9709 (0.9670–0.9744) | 0.9678 (0.9627–0.9724) |
| detection overall mean |  |  | 0.9064 (-0.1155–1.6685) | 0.9332 (-0.2780–1.8650) |
| detection standard deviation |  |  | 0.9841 (0.5191–2.5646) | 1.2208 (0.6054–3.4820) |
| detection effect pool A |  |  | 0.2590 (0.1442–0.6173) | 0.3429 (0.2067–0.4271) |
| detection effect pool L |  |  | 0.1543 (0.1236–0.1846) | 0.3313 (0.2683–0.7296) |
| detection effect pool P |  |  | 0.7720 (0.5173–0.9364) | 0.3639 (0.1404–0.4676) |
| detection effect pool S |  |  | 1.3143 (1.2475–1.0996) | 1.5065 (1.1463–4.0551) |
| detection effect pool M |  |  | 1.6688 (1.2254–2.3550) | 1.7175 (1.4754–2.9824) |
| detection effect pool D |  |  | 2.1760 (1.7864–2.6334) | 2.4770 (1.8409–4.0946) |
